# Interacción entre los determinantes medioambientales y socioeconómicos para el riesgo para leishmaniasis cutánea en América Latina

**DOI:** 10.26633/RPSP.2021.49

**Published:** 2021-04-28

**Authors:** Ana Nilce S. Maia-Elkhoury, Daniel Magalhães Lima, Oscar Daniel Salomón, Lia Puppim Buzanovsky, Martha Idalí Saboyá-Díaz, Samantha Y.O.B. Valadas, Manuel J. Sanchez-Vazquez

**Affiliations:** 1 Organización Panamericana de la Salud Río de Janeiro Brasil Organización Panamericana de la Salud, Río de Janeiro, Brasil.; 2 Centro Panamericano de Fiebre Aftosa Organización Panamericana de la Salud Río de Janeiro Brasil Centro Panamericano de Fiebre Aftosa, Organización Panamericana de la Salud, Río de Janeiro, Brasil.; 3 Instituto Nacional de Medicina Tropical Puerto Iguazú Argentina Instituto Nacional de Medicina Tropical, Puerto Iguazú, Argentina.; 4 Organización Panamericana de la Salud Washington, D.C. Estados Unidos de América Organización Panamericana de la Salud, Washington, D.C., Estados Unidos de América.

**Keywords:** Leishmaniasis cutánea, análisis por conglomerados, América Latina, Leshmaniasis, cutaneous, cluster analysis, Latin America, Leishmaniose cutânea, análise por conglomerados, América Latina

## Abstract

**Objetivo.:**

Determinar y caracterizar áreas de riesgo potencial de la ocurrencia de leishmaniasis cutánea (LC) en América Latina (AL).

**Método.:**

Estudio observacional ecológico con unidades de observación definidas por municipios con transmisión de LC entre 2014-2018. Se utilizaron variables medioambientales y socioeconómicas disponibles para al menos 85% de los municipios, combinados en una sola base de datos, a través del *software* R. Se combinó la metodología de análisis de componentes principales con un análisis de conglomerados jerárquicos para la formación de conglomerados de municipios en función de su similitud. Se estimó el V-test para definir la asociación positiva o negativa de las variables con los conglomerados y separación por divisiones naturales para determinar cuáles contribuyeron más a cada conglomerado. Se incorporaron los casos para atribuir el riesgo de LC para cada conglomerado.

**Resultados.:**

Se incluyeron en el estudio 4 951 municipios con transmisión de LC (36,5% del total en AL) y se definieron siete conglomerados por su asociación con 18 variables medioambientales y socioeconómicas. El riesgo histórico de LC se asocia de manera positiva y en forma decreciente con los conglomerados Amazónico, Andino y Sabana; y de manera negativa con los conglomerados Boscoso/perenne, Boscoso/cultivo y Boscoso/poblado. El conglomerado Agrícola no reveló ninguna asociación con los casos de LC.

**Conclusiones.:**

El estudio permitió identificar y caracterizar el riesgo de LC por conglomerados de municipios y conocer el patrón propio epidemiológico de distribución de la transmisión, lo que proporciona a los gestores una mejor información para las intervenciones intersectoriales para el control de la LC.

## Abstract (en)

**Objective.:**

Determine and characterize areas at potential risk for the occurrence of cutaneous leishmaniasis (CL) in Latin America.

**Method.:**

Ecological observational study with observation units defined by municipalities with CL transmission during 2014-2018. Environmental and socioeconomic variables available for at least 85% of municipalities were combined in a single database, using R software. Principal component analysis was combined with hierarchical cluster analysis for the formation of clusters of municipalities according to their similarity. The V-test was used to define positive or negative association of variables with clusters and separation by natural divisions to determine which contributed more to each cluster. Cases were included to attribute CL risk for each cluster.

**Results.:**

The study included 4 951 municipalities with CL transmission (36.5% of municipalities in Latin America); seven clusters were defined by their association with 18 environmental and socioeconomic variables. Historical risk of CL is associated positively and in descending order with the Amazonian, Andean, and Savanna clusters; and negatively with the Forest/perennial, Forest/cultivated, and Forest/populated clusters. The Agricultural cluster showed no association with CL cases.

**Conclusions.:**

The study made it possible to identify and characterize CL risk by clusters of municipalities and to understand the characteristic epidemiological distribution patterns of transmission, providing program managers with better information for intersectoral interventions to control CL.

## RESUMO

**Objetivo.:**

Determinar e caracterizar as áreas de risco de ocorrência de leishmaniose cutânea na América Latina.

**Método.:**

Estudo observacional ecológico com unidades de observação definidas por municípios com transmissão de leishmaniose cutânea entre 2014 e 2018. Foram usadas as variáveis ambientais e socioeconômicas disponíveis em 85% ou mais dos municípios, reunidas em uma única base de dados com o uso do software R. A metodologia de análise de componentes principais foi combinada a uma análise de conglomerados com agrupamento hierárquico para formar conglomerados de municípios por semelhança. O teste V foi usado para estabelecer a associação (positiva ou negativa) das variáveis com os conglomerados e uma separação por divisões naturais foi usada para determinar as variáveis que mais contribuíram em cada conglomerado. Os casos foram incluídos para avaliar o risco de leishmaniose cutânea em cada conglomerado.

**Resultados.:**

A amostra do estudo compreendeu 4.951 municípios com transmissão de leishmaniose cutânea (36,5% do total na América Latina). Foram definidos sete conglomerados por apresentarem associação com 18 variáveis ambientais e socioeconômicas. Foi observada associação positiva e decrescente do risco histórico de leishmaniose cutânea com os conglomerados Amazônico, Andino e Savana e negativa com os conglomerados Mata/perene, Mata/cultivo e Mata/povoado. O conglomerado Agrícola não demonstrou associação com casos de leishmaniose cutânea.

**Conclusões.:**

Este estudo permitiu identificar e caracterizar o risco de leishmaniose cutânea por conglomerados de municípios e conhecer o padrão epidemiológico de distribuição da transmissão da doença, oferecendo às autoridades dados melhores para subsidiar as intervenções intersetoriais para o controle da leishmaniose cutânea.

La leishmaniasis cutánea (LC) es una enfermedad causada por más de 20 especies de parásitos del género *Leishmania* y se transmite a través de vectores de la familia Psychodidae (1). En la Región de las Américas es una zoonosis, y se ha encontrado *Leishmania sp* como agente infeccioso en distintas especies de mamíferos silvestres (2). Es un problema de salud pública, con distribución mundial en 88 países. En el continente americano es endémica en 18 países, con un registro anual de aproximadamente 46 000 casos con distintas manifestaciones clínicas, de las cuales la LC localizada es la más frecuente (3,4).

La LC sigue siendo una de las enfermedades infecciosas desatendidas (EID) de gran importancia debido a su fuerte asociación con la pobreza (3,5). La lucha contra las leishmaniasis tiene estrecha relación con los objetivos de desarrollo sostenible (ODS), en especial el ODS 3 (salud y bienestar) y aquellos que son posibles determinantes para la ocurrencia de la enfermedad como, por ejemplo, el ODS 1 (reducción de la pobreza), el ODS 2 (promover la agricultura sostenible), el ODS 6 (acceso a agua y saneamiento), el ODS 8 (crecimiento económico y el empleo pleno y productivo), el ODS 13 (cambio climático) y el ODS 15 (protección de los ecosistemas terrestres). Por lo tanto, se requiere un abordaje integrado, tanto programático como multisectorial, para poner en marcha políticas de salud efectivas y reducir el daño en las poblaciones afectadas (6).

La ocurrencia de la LC está determinada por la exposición de los seres humanos (en general, por actividades económicas y sociales) en contextos donde existen las condiciones climáticas y ecológicas para la presencia de los vectores, parásitos y reservorios involucrados en la transmisión. Estos contextos modulan el aumento del nivel de la exposición humana (7), presionan la adaptación de especies de vectores a nichos ecológicos nuevos, creados por intervenciones antropógenas en el ambiente favorables para la proliferación de los vectores (8), e incrementan las interacciones entre reservorios y parásitos; de esta manera, contribuyen al mantenimiento del ciclo de transmisión de la enfermedad (2).

Se ha documentado la relación estrecha entre el cambio climático y la emergencia y reemergencia de algunas EID, incluidas las leishmaniasis, en varios lugares del mundo (9). Por ejemplo, estudios recientes mostraron la probable expansión futura de hábitats para vectores de leishmaniasis cutánea en Sudamérica (10) y en Brasil (11). Asimismo, se ha documentado el aumento del riesgo de exposición y ocurrencia de las leishmaniasis en poblaciones que viven en condiciones de pobreza, relacionadas principalmente con las características de las viviendas (proximidad de bosques, condiciones que favorecen la entrada del vector en el domicilio y hacinamiento, entre otras), bajas coberturas de acceso a servicios de agua y saneamiento, analfabetismo y dificultades para comprender los procesos de transmisión y prevención, entre otros (5). En un estudio sobre factores ambientales y socioeconómicos determinantes de la ocurrencia de la LC en Brasil, se encontró que la temperatura, la presencia de bosques, los tipos de vegetación, el grado de urbanización, el saneamiento, el desarrollo humano, la renta, la población y las áreas rurales, los hábitos culturales, la ocupación profesional, las actividades agropecuarias, la deforestación y la minería fueron los más relevantes (12). El presente estudio tiene el objetivo de determinar y caracterizar las áreas de riesgo potencial de la ocurrencia de LC en América Latina (AL) mediante el uso de variables relacionadas con los determinantes medioambientales y socioeconómicos.

## MATERIALES Y MÉTODOS

### Tipo, período y población de estudio

Se trata de un estudio observacional ecológico. Las unidades de observación fueron los 4 951 municipios de AL (36,5% municipios del total de municipios en AL) donde hubo transmisión de LC durante los años 2014 a 2018, según registros del sistema de información regional (SisLeish) (13). Este sistema consolida cada año los datos de ocurrencia de LC por municipio notificados por los ministerios de salud (13).

### Estrategia de análisis

Primero se identificaron aquellas variables medioambientales y socioeconómicas que pudieran estar asociadas con el riesgo de LC. A continuación, a través de una metodología de análisis multivariados, se crearon conglomerados jerárquicos de municipios que reunían características similares en función de las variables medioambientales. Este análisis multivariado se repitió con la inclusión de las variables socioeconómicas. Luego, se evaluaron los resultados de los dos enfoques de conglomerados jerárquicos, con o sin variables socioeconómicas, para decidir cuál caracterizaba mejor el riesgo de LC. Por último, se exploró la distribución de riesgo para cada uno de los conglomerados, para caracterizarlos de acuerdo con el riesgo histórico.

### Variables medioambientales y socioeconómicas

Con base en los principales determinantes para la LC y para otras EID (12,14), se realizó una búsqueda de datos que estuvieran disponibles para al menos 85% de los municipios con transmisión de la enfermedad en AL. Se encontraron datos matriciales de temperatura, altitud, precipitación, presencia de bosques, tipos de vegetación, actividades agropecuarias y minería (14–17), y datos tabulares de saneamiento, agua, hacinamiento y analfabetismo compilados por un estudio previo en el que se usaron datos de los censos de población y vivienda de los países (18). En el cuadro 1 se describen las variables incluidas en el estudio, las fuentes y los metadatos. A través de los límites geográficos de los municipios y, con el uso del paquete *exactextractr* (19), del *software* R (20), se compilaron los datos matriciales y se los combinó en una sola base de datos junto con los datos tabulares.

### Análisis multivariado por conglomerados jerárquicos

Para caracterizar los municipios en función de las variables identificadas anteriormente y crear los conglomerados de municipios, se combinó la metodología de análisis de componentes principales y se mantuvieron las cinco primeras dimensiones, con un análisis de conglomerados jerárquicos (21–23). Los conglomerados son un conjunto de municipios con características similares entre sí pero que, al mismo tiempo, mantienen la mayor diferenciación posible con municipios incluidos en otros conglomerados. En este procedimiento, el número de conglomerados resultantes es un parámetro flexible definido por el analista (21). Por ello, se probaron de cinco a diez posibilidades y se observó que siete conglomerados era un número adecuado para lograr una discriminación de acuerdo con los objetivos del estudio.

Se hizo un análisis multivariado por conglomerados jerárquicos para las variables medioambientales (enfoque A) y otro para el análisis conjunto de las variables medioambientales y las socioeconómicas (enfoque B).

Como resultado, se obtuvo una discriminación exhaustiva de conglomerados estadísticos (no espaciales) de municipios en función de su similitud con respecto al mayor o menor grado de participación de las variables de riesgo. Para ello, se estimó el V-test, que representa la asociación positiva o negativa de las variables con los conglomerados, y se utilizó como un indicador de la importancia relativa de ese factor en el conglomerado. Para determinar las variables que más contribuyeron a cada conglomerado, se utilizó una separación por divisiones naturales (24,25). Así, los valores absolutos de los V-test para cada conglomerado se discriminaron en cinco grupos y se seleccionaron las variables con los valores más altos para interpretar las características principales en cada uno.

**CUADRO 1.** Descripción de las variables incluidas en el estudio, fuentes y metadatos

Tipo	Factor relacionado	Determinante	Variable	Unidad	Abreviatura	Fuente	Año	Formato	Resolución
Medioambientales	Factores ambientales	Temperatura	Temperatura mínima mensual media	ºC	TEMPMINMED	WorldClim (14)	2010-2018	Matricial	2,5 arcos de minuto
			Temperatura máxima mensual media	ºC	TEMPMAXMED	WorldClim (14)	2010-2018	Matricial	2,5 arcos de minuto
		Precipitación	Precipitación mensual media	mm	PRECMED	WorldClim (14)	2010-2018	Matricial	2,5 arcos de minuto
		Altitud	Elevación media	metros	ELEVMED	WorldClim (14)	2000	Matricial	2,5 arcos de minuto
		Cobertura forestal de cualquier tipo	Cobertura forestal	% del territorio	COBFORESTA	FAO (15)	2007	Matricial	5 arcos de minuto
		Tipos de vegetación	Cobertura terrestre con bosques perennes o semicaducifolios	% del territorio	COBPERENNE	ESA (16)	2009	Matricial	10 arcos de segundo
			Cobertura terrestre con bosques caducifolios cerrados	% del territorio	COBCADUCIF	ESA (16)	2009	Matricial	10 arcos de segundo
			Cobertura terrestre con arbustos (<5 m de altura)	% del territorio	COBARBUSTO	ESA (16)	2009	Matricial	10 arcos de segundo
Socioeconómicas	Factores laborales y de incremento de exposición al vector	Minería	Actividades de minería	% del territorio	MINERIA	USGS (17)	2011	Vectorial	Transformado para matricial en superficie de densidad matricial de 10 km de radio
		Actividades agropecuarias	Cobertura terrestre con plantación con riesgo por lluvia	% del territorio	COBPLANLLUV	ESA (16)	2009	Matricial	10 arcos de segundo
			Cobertura terrestre con preponderancia de plantación en relación a vegetación	% del territorio	COBPLANVEG	ESA (16)	2009	Matricial	10 arcos de segundo
			Cobertura terrestre con preponderancia de vegetación sobre plantación	% del territorio	COBVEGPLAN	ESA (16)	2009	Matricial	10 arcos de segundo
			Cobertura terrestre con preponderancia de bosque sobre pastos	% del territorio	COBBOSPAST	ESA (16)	2009	Matricial	10 arcos de segundo
			Cobertura terrestre con agricultura tropical	% del territorio	COBAGRITROP	ESA (16)	2009	Matricial	10 arcos de segundo
	Factores de las personas y las viviendas	Analfabetismo	Tasa de analfabetismo	% de la población	ANALFAB	Censos de los países (18)	Variable^a^	Tabular	Municipio^b^
		Acceso al agua	Acceso inadecuado al agua	% de la población	AGUAINAD	Censos de los países (18)	Variable^a^	Tabular	Municipio^b^
		Saneamiento	Acceso inadecuado al saneamiento	% de la población	SANEINAD	Censos de los países (18)	Variable^a^	Tabular	Municipio^b^
		Hacinamiento	Hacinamiento (habitaciones con más de 3 personas)	% de las viviendas	HACINAM	Censos de los países (18)	Variable^a^	Tabular	Municipio^b^

a Datos compilados por un estudio previo en el que se usaron los datos del censo de población y vivienda más reciente de cada país publicados entre los años 2000 y 2012 (18).

b Para efectos del estudio, se denominó de forma genérica municipio a las provincias, distritos, municipios, cantones, etc., según la nomenclatura y estructura en cada país (18).

FAO, Organización de las Naciones Unidas para la Alimentación y la Agricultura (por su sigla en inglés); ESA, Agencia Espacial Europea (por su sigla en inglés); USGS, Servicio Geológico de Estados Unidos (por su sigla en inglés).

***Fuente:*** elaborado por los autores con los resultados del estudio y con permiso de los autores del estudio original del cual se obtuvieron los datos de los factores de las personas y las viviendas*.*

### Discriminación del enfoque más apropiado

Para decidir cuál enfoque (es decir, con o sin variables socioeconómicas) caracterizaba mejor el riesgo de LC, se evaluaron los dos resultados de conglomerados jerárquicos. Así se compararon las variables que más contribuyeron a la caracterización de los conglomerados con uno y otro enfoque para determinar la relevancia de incorporar las variables socioeconómicas, y su distribución geográfica.

### Determinación del riesgo por conglomerado

Una vez definido el enfoque, y con los resultados finales de los conglomerados, se procedió a identificarlos según su nivel de riesgo. Para ello, se utilizaron los casos de LC notificados en el SisLeish entre el 2014 y el 2018 (13) y se incorporaron como variable ilustrativa al análisis multivariado por conglomerados jerárquicos. Si bien esta variable no participó en la delimitación de los conglomerados, la asociación de riesgo de LC con el conglomerado se presenta en función del resultado de su V-test (23).

## RESULTADOS

De los 4 985 municipios con transmisión de LC en 16 países de AL, se incluyeron en el estudio los 4 951 municipios para los cuales se encontró suficiente información de las 18 variables seleccionadas.

### Resultado de la discriminación por el enfoque más apropiado

**FIGURA 1.**
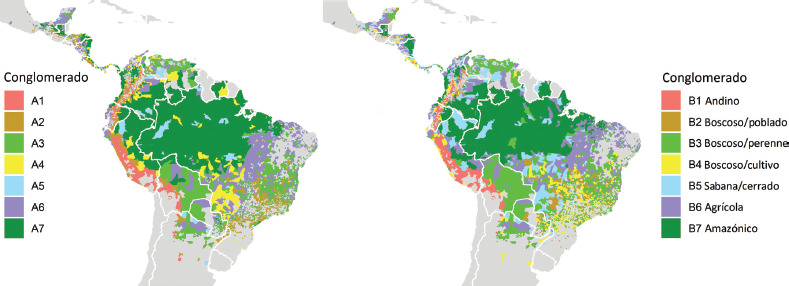
Distribución espacial de conglomerados formados a través de variables medioambientales en municipios con transmisión de LC, enfoque A (izquierda) y de variables medioambientales y socioeconómicos en municipios con transmisión de LC entre 2014 y 2018 en América Latina, enfoque B (derecha)

Como resultado de los análisis multivariados por conglomerados jerárquicos de municipios, se identificaron siete conglomerados definidos por su asociación con variables medioambientales, enfoque A (cuadro 2 y figura 1), y siete definidos por su asociación con variables medioambientales y socioeconómicas, enfoque B (cuadro 3 y figura 1). La comparación entre los dos enfoques reveló que la inclusión de las variables socioeconómicas permitió una discriminación más exhaustiva de los conglomerados, y una interpretación más integral del riesgo de transmisión de la LC. Esto puede observarse claramente en la discriminación alcanzada en la mitad sur de Brasil con el enfoque B (versus el A). Así, se observa que las variables socioeconómicas relacionadas con el saneamiento, la educación, el agua potable y el hacinamiento son clave para la formación de los conglomerados Boscoso/cultivo y Amazónico. Por otro lado, las variables socioeconómicas relacionadas con el aspecto laboral y el nivel de exposición, como las actividades agropecuarias, son clave para la formación de los conglomerados Boscoso/poblado y Boscoso perenne. Estos conglomerados contribuyen a diferenciar las áreas donde existe riesgo de ocurrencia de LC y a conocer los factores que contribuyen a dicho riesgo. El enfoque B, con variables medioambientales y socioeconómicas, fue considerado el más apropiado por ser el que aporta más información en la caracterización del riesgo de ocurrencia de la LC.

**CUADRO 2.** Variables^a^ ambientales con mayor peso según el V-test en cada conglomerado

Conglomerado	Variable	V-test	Media en el conglomerado	Media general	DE en el conglomerado	DE general
A1	ELEVMED	53,7	2899,8	644,6	657,7	701,8
	COBFORESTA	-15,1	17,3	39,9	14,4	25,1
	TEMPMINMED	-45,2	7,0	18,0	3,8	4,1
	TEMPMAXMED	-46,1	18,6	28,6	2,7	3,6
A2	ELEVMED	18,1	1032,6	644,6	460,5	701,8
	COBPERENNE	-11,3	0,2	0,3	0,2	0,3
	TEMPMAXMED	-24,2	25,9	28,6	1,8	3,6
	TEMPMINMED	-24,2	15,0	18,0	1,6	4,1
A3	COBPERENNE	55,5	0,7	0,3	0,2	0,3
	COBCADUCIF	-14,4	0,0	0,0	0,0	0,1
	PRECMED	-20,4	102,8	131,2	30,9	56,9
	COBARBUSTO	-22,3	0,0	0,1	0,0	0,1
A4	COBARBUSTO	52,4	0,4	0,1	0,1	0,1
	PRECMED	6,1	148,5	131,2	45,2	56,9
	COBPERENNE	-17,3	0,1	0,3	0,1	0,3
A5	COBCADUCIF	54,4	0,3	0,0	0,1	0,1
	COBARBUSTO	11,3	0,2	0,1	0,1	0,1
	COBPERENNE	-8,1	0,2	0,3	0,1	0,3
A6	TEMPMAXMED	23,8	30,8	28,6	1,8	3,6
	TEMPMINMED	20,1	20,0	18,0	2,0	4,1
	PRECMED	-14,7	110,3	131,2	35,5	56,9
	ELEVMED	-18,4	321,1	644,6	216,7	701,8
	COBPERENNE	-20,4	0,2	0,3	0,1	0,3
A7	COBFORESTA	41,0	73,3	39,9	18,7	25,1
	PRECMED	37,8	201,1	131,2	70,5	56,9
	TEMPMINMED	27,7	21,6	18,0	1,6	4,1
	TEMPMAXMED	22,4	31,2	28,6	1,5	3,6

a Todas las variables presentadas tuvieron un valor de p <0,001.

DE, desviación estándar.

***Fuente:*** elaborado por los autores con los resultados del estudio.

***Fuente:*** elaborada por los autores con los resultados del estudio.

**FIGURA 2.**
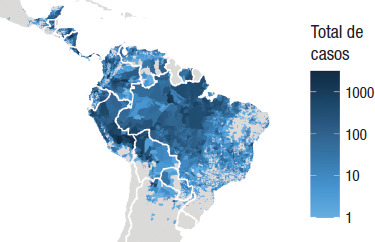
Distribución espacial de los casos de LC en América Latina notificados entre 2014 y 2018 en escala logarítmica de base 10

La distribución de casos de LC por municipio en el período 2014-2018 se presenta en la figura 2. El riesgo histórico de LC aparece asociado de manera positiva en forma decreciente con los conglomerados Amazónico, (V-test: 11,44), Andino (V-test: 3,25) y Sabana (V-test: 3,08); y negativamente asociado con el conglomerado Boscoso/perenne, (V-test: -5,52), Boscoso/cultivo (V-test: -4,66) y Boscoso/poblado (V-test: -3,35). El conglomerado Agrícola no reveló ninguna asociación con los casos de LC.

## DISCUSIÓN

La Organización Mundial de la Salud incluyó las leishmaniasis entre las 20 enfermedades que afectan de manera desproporcionada a las poblaciones que viven en condiciones de pobreza, sobre todo en áreas tropicales y subtropicales (26). Entender los factores asociados a la ocurrencia de la LC ha sido objeto de múltiples estudios, incluidas algunas aproximaciones para el análisis de los factores climáticos y condiciones ambientales en las Américas (27).

En el presente estudio, la incorporación de variables relacionadas con las condiciones de vida de las personas (acceso a agua potable, saneamiento básico, hacinamiento, educación, actividad agrícola y minería) contribuyó a una mejor caracterización de los conglomerados de municipios con riesgo de transmisión de la LC en América Latina al combinarlas con las variables ambientales relacionadas con la temperatura, la precipitación, la elevación y el tipo de vegetación.

Aunque para otras EID estas variables de condiciones de vida suelen incluirse con mayor frecuencia en el análisis de riesgo de la transmisión, como en las geohelmintiasis o el tracoma (18,28), en una escala más desagregada como la municipal, no es frecuente la inclusión específica para el análisis de los factores asociados con la transmisión de la LC. Esta aproximación analítica a escala municipal permitió caracterizar siete conglomerados de transmisión de la LC, por lo que es una herramienta útil para apoyar el diseño para el desarrollo de las acciones de vigilancia e intervenciones de prevención y control, centradas en los contextos sociales, económicos, epidemiológicos y ambientales de las comunidades afectadas.

En las áreas con casos autóctonos de LC, los factores ambientales posibilitan el ciclo parasitario y su continuidad en el tiempo, mientras que las variables sociales determinan la probabilidad de transmisión a los seres humanos al modular su exposición y vulnerabilidad, y ambas se integran en el contexto socioambiental particular de cada foco (12,29). Por ello, las características generales de cada conglomerado (cuadro 3) permiten identificar diferentes escenarios de riesgo de brote según la interacción humana con el ambiente. Estos escenarios son útiles para hacer un mejor diseño de acciones para el monitoreo, generar alertas tempranas y definir las actividades de mitigación, aún en las áreas sin casos.

El conglomerado Amazónico, el de mayor asociación con casos de LC, presenta focos de incidencia elevada por proximidad al ciclo silvestre de la LC. Esto se refleja, por ejemplo, en la ocurrencia de casos en los municipios de Tumaco (Colombia) y en Waslala, Rancho Grande, San José de Bocay y Cuá (Nicaragua). Sin embargo, en este conglomerado también hay focos de menor incidencia asociados a la desforestación con asentamientos humanos dispersos. En este conglomerado, los brotes y casos aislados se originan por “intromisión” de los seres humanos en la selva durante actividades extractivas, militares, de investigación y recreativas. Sin embargo, también hay intromisión de los seres humanos por actividades ilegales, lo que causa subregistro de casos y dificultad para localizar el sitio preciso de transmisión. En los campamentos humanos provisorios o semipermanentes, se incrementa el riesgo de LC por hacinamiento, exposición en bosques riparios y el nivel de analfabetismo (30,31). Este es un conglomerado con escenarios epidemiológicos muy dinámicos, que requieren monitoreo continuo debido al cambio masivo del uso de la tierra, el crecimiento de ciudades colindantes con la selva y los incendios a gran escala, lo que genera presión para la dispersión y adaptación del ciclo de transmisión de la LC a ambientes antropizados (32,33).

**CUADRO 3.** Características generales de los conglomerados, variables^a^ ambientales y socioeconómicas con mayor peso^b^ según el V-test en cada uno

Conglomerado	N° de municipios	Características generales	Variable	V-test	Media del conglomerado	Media general	DE conglomerado	DE general
Andino	333	Valles interandinos, laderas de los Andes, presencia de minería y zonas con acceso inadecuado a agua	ELEVMED	54,2	2656,5	644,6	769,3	701,8
			MINERIA	25	21,2	5,1	27,6	12,1
			TEMPMINMED	-44,8	8,4	18	4,3	4,1
			TEMPMAXMED	-46,8	19,6	28,6	3,2	3,6
Boscoso/poblado	311	Zonas con predominio de bosques, menor presencia de agricultura tropical y plantaciones. Presencia de áreas urbanas y comunidades en proceso de urbanización	BOSPASTO	54,5	0,1	0	0,1	0
			AGRITROP	-8,1	0,3	0,4	0,3	0,4
			PLANTACVEG	-14,3	0,1	0,2	0,1	0,2
Boscoso/perenne	1347	Áreas con cobertura de bosques perennes y extensa continuidad geográfica	COBPERENNE	55,3	0,7	0,3	0,2	0,3
			PLANTACLLUV	-22	0	0,1	0	0,1
			VEGPLANTAC	-27,8	0,1	0,2	0,1	0,1
			PLANTACVEG	-31,8	0,1	0,2	0,1	0,2
			AGRITROP	-33,7	0,1	0,4	0,2	0,4
Boscoso/cultivo	850	Áreas boscosas, cultivos tropicales y localidades de desarrollo medio, y parches remanentes de selva tropical	VEGPLANTAC	32,1	0,3	0,2	0,2	0,1
			AGRITROP	16,3	0,6	0,4	0,3	0,4
			TEMPMINMED	-14,3	16,2	18	2,3	4,1
			HACINAM	-15,6	0	0,1	0	0,1
			COBPERENNE	-15,7	0,2	0,3	0,2	0,3
			AGUAINAD	-16,7	0,1	0,2	0,1	0,2
			ANALFAB	-17,6	0,1	0,2	0,1	0,1
			SANEINAD	-17,9	0,1	0,2	0,1	0,2
Sabana (cerrado)	505	Coberturas arbustivas y bosques cerrados caducifolios En las márgenes del conglomerado Amazónico, reservas biológicas, o en valles al pie de selva tropical húmeda Zonas con deficiente acceso a agua, saneamiento y educación	COBARBUSTO	41	0,3	0,1	0,2	0,1
			COBCADUCIF	36,6	0,1	0	0,2	0,1
			COBPERENNE	-15,9	0,1	0,3	0,1	0,3
Agrícola	1066	Áreas con actividades agrícolas tropicales, como las áreas cultivadas por riesgo de lluvia, temperaturas medias elevadas y altitud baja, cultivos tropicales, y menor proporción de zonas boscosas y pasto	PLANTACLLUV	26,4	0,2	0,1	0,2	0,1
			PLANTACVEG	23,5	0,3	0,2	0,2	0,2
			TEMPMAXMED	23,4	30,9	28,6	2,1	3,6
			AGRITROP	23,3	0,7	0,4	0,3	0,4
			TEMPMINMED	22,4	20,4	18	2	4,1
			ANALFAB	20,2	0,2	0,2	0,1	0,1
			BOSPASTO	-11,9	0	0	0	0
			ELEVMED	-17,3	315,5	644,6	267,4	701,8
			COBPERENNE	-17,6	0,2	0,3	0,1	0,3
Amazónico	539	Gran cobertura vegetal, temperaturas medias y de precipitación alta que forman la cuenca amazónica y la selva tropical húmeda Presenta asociación alta con ocurrencia de casos de LC y un área geográfica extensa y continua Zona con acceso inadecuado al agua y al saneamiento, y con analfabetismo	HACINAM	35,3	0,2	0,1	0,1	0,1
			PRECMED	34,2	210,4	131,2	76,7	56,9
			AGUAINAD	32,7	0,4	0,2	0,2	0,2
			COBFORESTA	32,3	72,9	39,9	21,2	25,1
			SANEINAD	30,6	0,4	0,2	0,2	0,2
			TEMPMINMED	22,6	21,7	18	1,7	4,1
			ANALFAB	20,8	0,2	0,2	0,1	0,1
			TEMPMAXMED	16,2	31	28,6	1,7	3,6

a Los análisis de todas las variables ambientales y socioeconómicas que tuvieron significancia estadística con relación al V-test en cada conglomerado se detallan en la Información suplementaria.

b Todas las variables presentadas tuvieron un valor de *P* <0,001.

DE, desviación estándar; LC; leishmaniasis cutánea.

***Fuente:*** elaborado por los autores con los resultados del estudio.

***Fuente:*** elaborada por los autores con los resultados del estudio.

En el conglomerado Andino, las barreras físicas y las diferencias de altitud generan gran heterogeneidad de ecosistemas en áreas bastante reducidas, que resultan en la variedad de parásitos, vectores y reservorios asociadas a este conglomerado. Por ejemplo, la *L. peruviana* tiene transmisión endémica en comunidades del altiplano semiárido y hay focos epidémicos por *L. guyanensis* debido a intervenciones en el bosque lluvioso tropical de altura. En algunos municipios, como los de Tolima (Colombia), la transmisión suele ocurrir a los 1 000-2 000 msnm, aunque hay registros de transmisión en otras regiones a mayor altura, posiblemente favorecidos por el cambio climático. La minería, en este contexto, es una actividad de riesgo para la ocurrencia de LC en los seres humanos debido a que aumenta el contacto con los ciclos enzoóticos durante la actividad laboral y en los campamentos en los que se alojan, como ocurrió con la bartonelosis transmitida por Phlebotominae en la construcción del Ferrocarril Central de Perú. En este conglomerado, también ocurre transmisión epidémica o endémica, peridoméstica y doméstica en áreas recientemente deforestadas de localidades rurales para cultivos de subsistencia y explotaciones agrícolas, como el café en el municipio de Rovira (Colombia) (34,35).

Las áreas delimitadas por el conglomerado Sabana suelen ser zonas de transición, con presencia de comunidades rurales dispersas, como los municipios del estado de Pará (Brasil) o del Chocó (Colombia). En los focos de LC de intensidad mediana y sostenida, la distribución etaria y por sexo de los casos sugiere un componente de transmisión peridoméstica aún en asentamientos antiguos próximos al ciclo silvestre. Sin embargo, alteraciones socioambientales en “puntos calientes” pueden generar brotes de gran magnitud, como resultó por la explotación y transporte de gas en La Convención en Perú (36,37).

El conglomerado Agrícola no presentó, en este estudio, una asociación positiva o negativa con los casos registrados de LC. La tasa de analfabetismo caracteriza, una vez más, a la determinación social de las comunidades expuestas y su capacidad de manejo. Sin embargo, estos resultados no implican riesgo bajo de transmisión, ya que involucra municipios con importantes registros de LC como Teolândia, en el área cacaotera de Bahía (Brasil), regiones de influencia amazónica con actividades extractivas, y la expansión de la frontera agrícola como en Rio Branco (Brasil) y Tambopata, Madre de Dios (Perú) o Choluteca en Honduras donde se presenta LC atípica debida a *L. infantum*. Este conglomerado involucra también áreas con transmisión intermedia endémica o con brotes limitados, donde el riesgo peridoméstico se relaciona con la proximidad a cursos de agua, cría de animales y parches de vegetación como bambú y bananas, como en Sapucaia (Brasil) (38,39).

Los conglomerados Boscoso/cultivo, perenne y poblado, en dicho orden, presentaron asociación negativa con los casos registrados de LC, en una gradación coherente con el grado de intervención antrópica en el ambiente y tasas altas e intermedias de LC en pocos municipios. El conglomerado Boscoso/cultivo incluye comunidades establecidas, en tierras ya deforestadas en grandes extensiones, pero donde los remanentes de selva tropical y la permanencia del ciclo silvestre en las áreas que no son rentables para deforestar pueden generar “bordes” con transmisión peridoméstica sostenida y brotes locales importantes, como en comunidades rurales o rururbanas de Orán (Argentina), Falan (Colombia) o Ferreñafe (Perú), donde el aumento de casos se asoció a progresión de brotes epidémicos desde regiones vecinas a la colonización de vectores adaptados a los ambientes antrópicos o áreas de ecoturismo con incidencia de LC en poblaciones indígenas (40,41).

El conglomerado Boscoso/perenne, junto al Amazónico, presenta la mayor continuidad espacial y cantidad de municipios, principalmente en el Estado Plurinacional de Bolivia y la República Bolivariana de Venezuela aunque es, a su vez, el que incluye menos información demográfica. El conglomerado registra transmisión esporádica por incursión en áreas de fragmentación boscosa, sugerida por la mayor proporción de casos de LC en personas de sexo masculino, como en Chapare (Bolivia) o Campinápolis y Mato Grosso (Brasil). Esta fragmentación, según su magnitud, ambiente original y calidad de vegetación secundaria o cultivo comercial, puede generar riesgo microfocal al concentrar vectores y reservorios de LC o diluirse hasta extinguir su transmisión. Los procesos de desforestación acelerada, migración y colonización contribuyen a que los factores de exclusión social modulen el riesgo ambiental y la vulnerabilidad a los eventos climáticos extremos, como se observó en Talamanca (Costa Rica) (42,43).

El conglomerado Boscoso/poblado, que presenta asociación negativa con los casos de LC de mayor magnitud, incluye áreas urbanizadas y en proceso de urbanización no planificada en zonas con vegetación residual o secundaria y desforestaciones puntuales para permitir nuevos asentamientos, como los municipios de Barra do Garças (Brasil), Cimitarra (Colombia) y Othón Pompeyo Blanco (México) (44,45). La población expuesta a este riesgo periurbano incluye tanto aquella que ocupa zonas con rentabilidad baja de la tierra, ya fragilizada por las inequidades del sistema, como a emprendimientos inmobiliarios nuevos en relación con el “retorno a la naturaleza”, con recursos propios y mayor presencia mediática.

Las limitaciones del estudio son propias de las fuentes de datos disponibles para un análisis en escala de municipio, como son los censos poblacionales y de viviendas que, en algunos países tienen más de 15 años, o por no disponibilidad de datos de calidad o de variables ambientales y socioeconómicas, como por ejemplo de renta y diferenciación entre áreas urbanas y rurales. Otra limitación es que la escala de municipio es diferente a la escala operacional de foco de LC y la identificación de determinantes de brotes epidémicos a dicha escala, que son puntuales en tiempo y espacio. Por otra parte, en relación con la vigilancia y registro de casos de LC, el sitio de notificación puede ser diferente al sitio de transmisión (por ejemplo, casos que ocurren en soldados, migrantes forzados o migrantes estacionales), lo que requiere un sistema de comunicación fluida entre los sitios de producción de casos y de recepción y diagnóstico. También hay limitaciones y posibles sesgos debidos al subregistro por falta de accesibilidad y sensibilidad diagnóstica del sistema de salud, lo que potencia la vulnerabilidad de las poblaciones ya vulnerables.

## CONCLUSIONES

El estudio permitió identificar y caracterizar el riesgo de LC por conglomerados de municipios. Esto contribuyó a conocer mejor el patrón propio epidemiológico de distribución de la transmisión para proporcionar a los gestores de programas de leishmaniasis una mejor información para la vigilancia y control de la enfermedad. El análisis de conglomerados a partir de determinantes socioambientales a escala municipal y su asociación con riesgos focales de transmisión epidémica de la LC demuestra que se requieren acciones simultáneas desde múltiples sectores, no solo del sector salud, para el control y mitigación del impacto de enfermedades zoonóticas como la LC. Las variables involucradas en la caracterización de los conglomerados evidencian su relación directa con la agenda del desarrollo sostenible, por lo que resulta necesario que los gestores y profesionales de la salud involucrados en las acciones de vigilancia y control de leishmaniasis en América Latina planifiquen sus intervenciones teniendo en cuenta los determinantes y caracterización de los diferentes conglomerados, los factores de riesgo y la ocurrencia de enfermedad o su transmisión potencial en áreas que, por ahora, siguen silenciosas o sin registros de LC.

## Declaración.

Las opiniones expresadas en este manuscrito son únicamente responsabilidad de los autores y no reflejan necesariamente los de la *Revista Panamericana de Salud Pública* o la Organización Panamericana de la Salud.

## References

[1] 1. Organización Mundial de la Salud. Global leishmaniasis update, 2006-2015: a turning point in leishmaniasis surveillance. Weekly epidemiological record N.° 38. 2017;92:557-572. Disponible en: https://www.who.int/leishmaniasis/resources/who_wer9238/en/

[2] 2. Roque ALR, Jansen AM. Wild and synanthropic reservoirs of Leishmania species in the Americas. Int J Parasitol Parasites Wildl. 2014;3(3):251-262. Doi: 10.1016/j.ijppaw.2014.08.004 10.1016/j.ijppaw.2014.08.004 PMC4241529 25426421

[3] 3. World Health Organization. Global leishmaniasis surveillance, 2017-2018, and first report on 5 additional indicators. Weekly epidemiological record N.° 25. 2020; 95:265-280. Disponible en: https://www.who.int/publications/i/item/who-wer9525

[4] 4. Organización Panamericana de la Salud. Leishmaniasis: informe epidemiológico en las Américas. [Internet]. Washington, D.C.: OPS; 2019 [consultado el 05 de octubre del 2020]. Disponible en: https://iris.paho.org/handle/10665.2/51739

[5] 5. Alvar J, Yactayo S, Bern C. Leishmaniasis and poverty. Trends Parasitol. 2006;22(12):552-557. Doi: 10.1016/j.pt.2006.09.004. 10.1016/j.pt.2006.09.004 17023215

[6] 6. Organización Mundial de la Salud. Health in 2015: from MDGS, millennium development goals, to SDGS, sustainable development goals. [Internet]. Ginebra: OMS; 2015 [consultado el 05 de octubre 2020]. Disponible en: https://apps.who.int/iris/handle/10665/200009

[7] 7. Shaw J. The leishmaniases, survival and expansion in a changing world: a mini-review. Mem Inst Oswaldo Cruz. 2007;102(5):541-547. 10.1590/s0074-02762007000500001 17899628

[8] 8. Rangel EF, Carvalho BM, Costa SM, Lainson R, Shaw JJ. Sand fly vectors of American cutaneous leishmaniasis in Brazil. 1° Ed. Rio de Janeiro: Springer, Cham; 2018:341-380.

[9] 9. El-Sayed A, Kamel M. Climatic changes and their role in emergence and re-emergence of diseases. Environ Sci Pollut Res Int. 2020;27(18):22336-22352. Doi: 10.1007/s11356-020-08896-w. 10.1007/s11356-020-08896-w PMC7187803 32347486

[10] 10. Carvalho BM, Rangel EF, Ready PD, Vale M.M. Ecological niche modelling predicts southward expansion of Lutzomyia (Nyssomyia) flaviscutellata (Diptera: Psychodidae: Phlebotominae), vector of Leishmania (Leishmania) amazonensis in South America, under climatecChange. PLoS ONE. 2015;10(11):e0143282. Doi: https://doi.org/10.1371/journal.pone.0143282. 10.1371/journal.pone.0143282 PMC4664266 26619186

[11] 11. Costa SM, Cordeiro LP, Rangel EF. Environmental suitability for Lutzomyia (Nyssomyia) whitmani (Diptera: Psychodidae: Phlebotominae) and the occurrence of American cutaneous leishmaniasis in Brazil. Parasites & Vectors. 2018;11(155):e0143282. 10.1186/s13071-018-2742-7 PMC5842654 29514680

[12] 12. Buzanovsky LP, Sanchez-Vazquez MJ, Maia-Elkhoury ANS, Werneck GL. Major environmental and socioeconomic determinants of cutaneous leishmaniasis in Brazil: a systematic literature review. Rev Soc Bras Med Trop. 2020;53:e20190291. Doi: https://doi.org/10.1590/0037-8682-0291-2019 10.1590/0037-8682-0291-2019 PMC7269534 32491100

[13] 13. Maia-Elkhoury ANS, Valadas SYOB, Puppim-Buzanovsky L, Rocha F, Sanchez-Vazquez MJ. SisLeish: a multi-country standardized information system to monitor the status of Leishmaniasis in the Americas. PLoS Negl Trop Dis. 2017;11(9):e0005868. Doi: https://doi.org/10.1371/journal.pntd.0005868 10.1371/journal.pntd.0005868 PMC5600406 28873400

[14] 14. Worldclim [Internet]. Historical climate data. [actualizado en enero del 2020; consultado el 19 junio del 2020]. Disponible en: https://www.worldclim.org/data/worldclim21.html

[15] 15. Organización de las Naciones Unidas para la Alimentación y la Agricultura [Internet]. Occurence of forest [actualizado en mayo del 2007; consultado el 19 de junio del 2020]. Disponible en: http://www.fao.org:80/geonetwork/srv/en/resources.get?id=14066&fname=Map5_1.zip&access=private

[16] 16. Agencia Espacial Europea [ Internet]. GlobCover 2009 [actualizado en diciembre del 2010; consultado el 19 de junio del 2020]. Disponible en: http://due.esrin.esa.int/page_globcover.php

[17] 17. U.S Geological Survey [Internet]. Mineral resources online spatial sata [consultado el 19 de junio del 2020]. Disponible en: https://mrdata.usgs.gov/#mineral-resources

[18] 18. Saboyá-Díaz MI, Betanzos-Reyes AF, West SK, Muñoz B, Castellanos LG, Espinal M. Trachoma elimination in Latin America: prioritization of municipalities for surveillance activities. Rev Panam Salud Publica. 2019;43:e93. Doi: https://doi.org/10.26633/RPSP.2019.93 10.26633/RPSP.2019.93 PMC7919757 33659029

[19] 19. Baston D. Exactextractr: fast extraction from raster datasets using polygons. R package version 0.3.0. [consultado el 05 de agosto del 2020]. Disponible en: https://CRAN.R-project.org/package=exactextractr. 2020.

[20] 20. R Core Team [Internet]. R: a language and environment for statistical computing. R Foundation for Statistical Computing, Vienna, Austria [consultado el 04 de agosto del 2020]. Disponible en: https://www.R-project.org/

[21] 21. Husson F, Josse J, Pagès J. Principal component methods - hierarchical clustering - partitional clustering: why would we need to choose for visualizing data? Tech Rep Appl Math Dep. 2010:1-17.

[22] 22. Husson F. Exploratory multivariate analysis by example using R. J Stat Softw. 2011;40.

[23] 23. Lë S, Josse J, Husson F. FactoMineR: an R package for multivariate analysis. J Stat Softw. 2008;25(1):1-18. Doi: 10.18637/jss.v025.i01

[24] 24. Armstrong MP, Xiao N, Bennett DA. Using genetic algorithms to create multicriteria class intervals for choropleth maps. Ann Assoc Am Geogr. 2003;93(3):595-623. Doi: https://doi.org/10.1111/1467-8306.9303005

[25] 25. Jenks G. Optimal data classification for choropleth maps. Kansas: University Dept. of Geography-Meteorology: 1977.

[26] 26. Organización Mundial de la Salud. Ending the neglect to attain the sustainable development goals: a road map for neglected tropical diseases 2021-2030. Ginebra: OMS; 2020. Disponible en: https://www.who.int/neglected_diseases/resources/who-ucn-ntd-2020.01/en/

[27] 27. Pérez-Flórez M, Ocampo CB, Valderrama-Ardila C, Alexander N. Spatial modeling of cutaneous leishmaniasis in the Andean region of Colombia. Mem Inst Oswaldo Cruz. 2016;111(7):433-442. Doi: https://doi.org/10.1590/0074-02760160074 10.1590/0074-02760160074 PMC4957495 27355214

[28] 28. Colston J, Saboyá M. Soil-transmitted helminthiasis in Latin America and the Caribbean: modelling the determinants, prevalence, population at risk and costs of control at sub-national level. Geospat Health. 2013;7(2):321-40. Doi: 10.4081/gh.2013.90 10.4081/gh.2013.90 23733294

[29] 29. Salomon OD. Instructions on how to make an outbreak of American cutaneous leishmaniasis. J Trop Med Health. 2019; 3:146. doi: 10.29011/2688-6383.000146

[30] 30. Chavy A, Nava AFD, Luz SLB, Ramírez JD, Herrera G, Dos Santos TV, et al. Ecological niche modelling for predicting the risk of cutaneous leishmaniasis in the neotropical moist forest biome. PLoS Negl Trop Dis. 2019;13(8):e0007629. Doi: 10.1371/journal.pntd.0007629 10.1371/journal.pntd.0007629 PMC6693739 31412022

[31] 31. Rodrigues MGA, Sousa JDB, Dias ÁLB, Monteiro WM, Sampaio VS. The role of deforestation on American cutaneous leishmaniasis incidence: spatial-temporal distribution, environmental and socioeconomic factors associated in the Brazilian Amazon. Trop Med Int Health. 2019;24(3):348-355. Doi: 10.1111/tmi.13196 10.1111/tmi.13196 30578585

[32] 32. Bonilla-Aldana DK, Suárez JA, Franco-Paredes C, Vilcarromero S, Mattar S, Gómez-Marín JE, et al. Brazil burning! What is the potential impact of the Amazon wildfires on vector-borne and zoonotic emerging diseases? A statement from an international experts meeting. Travel Med Infect Dis. 2019;31:101474. Doi: 10.1016/j.tmaid.2019.101474 10.1016/j.tmaid.2019.101474 31494225

[33] 33. Rebêlo JMM, Moraes JLP, Cruz GBV, Andrade-Silva J, Bandeira MDCA, Oliveira YNP, Santos CLCD. Influence of deforestation on the community structure of sand flies (Diptera: Psychodidae) in Eastern Amazonia. J Med Entomol. 2019;56(4):1004-1012. Doi: 10.1093/jme/tjz014. 10.1093/jme/tjz014 30887047

[34] 34. Hashiguchi Y, Gomez LEA, Cáceres AG, Velez LN, Villegas NV, Hashiguchi K, et al. Andean cutaneous leishmaniasis (Andean-CL, uta) in Peru and Ecuador: the vector Lutzomyia sand flies and reservoir mammals. Acta Trop. 2018; 178:264-275. Doi: 10.1016/j.actatropica.2017.12.008 10.1016/j.actatropica.2017.12.008 29224978

[35] 35. Hernández AM, Gutierrez JD, Xiao Y, Branscum AJ, Cuadros DF. Spatial epidemiology of cutaneous leishmaniasis in Colombia: socioeconomic and demographic factors associated with a growing epidemic. Trans R Soc Trop Med Hyg. 2019;113(9):560-568. doi: 10.1093/trstmh/trz043 10.1093/trstmh/trz043 31140567

[36] 36. Torres-Slimming P. Globalización, el proyecto Camisea y la salud de los matsiguengas. Rev Peru Med Exp Salud Publica. 2010;27(3):458-65. 10.1590/s1726-46342010000300021 21152741

[37] 37. Gutiérrez JD, Martínez-Vega R, Ramoni-Perazzi J, Diaz-Quijano FA, Gutiérrez R, Ruiz FJ, et al. Environmental and socio-economic determinants associated with the occurrence of cutaneous leishmaniasis in the northeast of Colombia. Trans R Soc Trop Med Hyg. 2017;111(12):564-571. Doi: 10.1093/trstmh/try011 10.1093/trstmh/try011 29509941

[38] 38. Guerra JAO, Guerra MGVB, Vasconcelos ZS, Freitas NS, Fonseca FR, da Silva Júnior RC, et al. Socioenvironmental aspects of the Purus Region - Brazilian Amazon: Why relate them to the occurrence of American tegumentary leishmaniasis? PLoS One. 2019;14(2): e0211785. Doi: https://doi.org/10.1371/journal.pone.0211785 10.1371/journal.pone.0211785 PMC6366772 30730951

[39] 39. de Bustamante MC, Pereira MJ, Schubach Ade O, da Fonseca AH. Epidemiological profile of cutaneous leishmaniasis in an endemic region in the State of Rio de Janeiro, Brazil. Rev Bras Parasitol Vet. 2009;18(3):34-40. Doi: https://doi.org/10.4322/rbpv.01803006 10.4322/rbpv.01803006 19772772

[40] 40. Quintana MG, Salomón OD, De Grosso MS. Distribution of phlebotomine sand flies (Diptera: Psychodidae) in a primary forest-crop interface, Salta, Argentina. J Med Entomol. 2010;47(6):1003-10. Doi: https://doi.org/10.1603/ME09072 10.1603/me09072 21175047

[41] 41. Zúñiga PM. Dimensión sociocultural de la leishmaniasis cutánea. Estados Unidos de América: Editorial Académica Española; 2015.

[42] 42. Chaves LF, Cohen JM, Pascual M, Wilson ML. Social exclusion modifies climate and deforestation impacts on a vector-borne disease. PLoS Negl Trop Dis. 2008;2(1):e176. Doi: https://doi.org/10.1371/journal.pntd.0000176 10.1371/journal.pntd.0000176 PMC2238711 18265876

[43] 43. Eid D, Guzman-Rivero M, Rojas E, Goicolea I, Hurtig AK, Illanes D, et al. Risk factors for cutaneous leishmaniasis in the rainforest of Bolivia: a cross-sectional study. Trop Med Health. 2018; 46:9. Doi: 10.1186/s41182-018-0089-6 10.1186/s41182-018-0089-6 PMC5902850 29692654

[44] 44. Gómez LE, Corredor A. Caracterización sociocultural y epidemiológica de un foco de leishmaniasis cutánea en Cimitarra, Santander. Rev Salud Publica 2000;2(3):261-271.

[45] 45. Cruz CB, da Silva MA, Afonso WBB. Perfil da população acometida por Leishmaniose tegumentar americana em Barra do Garças – MT. Revista Eletrônica Interdisciplinar. 2017;18(2):84-90.

